# Assessing risk of COVID-19 reinfection and complications in adults treated with stimulant medications: A retrospective study using electronic health records

**DOI:** 10.18103/mra.v12i9.5807

**Published:** 2024-09

**Authors:** Wen-Jan Tuan, Adriana Von Rago, Christopher Heron, Michael Partin, Kyle Burke, Grace Hwang, Aleksandra E. Zgierska

**Affiliations:** 1 Departments of Family and Community Medicine and Public Health Science, College of Medicine, Pennsylvania State University; 2 College of Medicine, Pennsylvania State University, Pennsylvania, USA; 3 Department of Family and Community Medicine, College of Medicine, Pennsylvania State University, Pennsylvania, USA; 4 Department of Family and Community Medicine, College of Medicine, Pennsylvania State University, Pennsylvania, USA; 5 Departments of Family and Community Medicine, Pennsylvania State University; 6 College of Medicine, Pennsylvania State University, Pennsylvania, USA; 7 Departments of Family and Community Medicine, Anesthesiology and Perioperative Medicine, and Public Health Science, Pennsylvania State University

**Keywords:** Prescription stimulant, COVID-19 reinfection, COVID-19 outcome

## Abstract

Despite advancement in vaccines and treatments for COVID-19 over time, many concerns remain about reinfection and waning immunity against COVID-19 and its variants, including among individuals treated with stimulants. The study aimed to evaluate the risk of COVID-19 reinfection and severe illness among adults prescribed stimulants compared to those not on stimulant therapy to elucidate the potential relationship of this therapy to the COVID-19 reinfection-related outcomes. A retrospective cohort study was conducted using the TriNetX database consisting of over 2,563,130 adults (age ≥18 years) with COVID-19 infection from January 1, 2020 to April 30, 2022. COVID-19 reinfection was defined as a new COVID-19 illness documented ≥ 30 days after the initial infection. Logistic regression was applied to assess the odds ratios (ORs) of COVID-19 reinfection and severe illness course (presence of emergence department (ED), hospitalization, and intensive care unit (ICU) care, mortality) within 30 days of reinfection, controlled for demographic and comorbid condition characteristics. Analysis revealed that adults who received at least three prescriptions for stimulants, compared to those without any stimulant prescriptions within six months prior to the initial COVID-19 illness, were more likely to be re-infected by COVID-19 (aOR=1.36, 95% confidence interval (CI): 1.32-1.41 ), but less likely to be treated in the ED (aOR=0.77, 95% CI: 0.72-0.82), hospital (aOR=0.72, 95% CI: 0.63-0.82), ICU (aOR=0.68, 95% CI: 0.53-0.89), or die (aOR=0.31, 95% CI: 0.15-0.64) within 30 days of reinfection (all p-values < 0.01). The study offers additional insights into the potential risks and benefits of prescription stimulants to aid in clinical decision making for treating COVID-19 reinfection in adults on stimulant therapy.

## 1. Introduction

The COVID-19 pandemic has disproportionately affected the populations experiencing chronic mental or physical health conditions.^1^ One of such populations comprises individuals on long-term stimulant therapy for attention-deficit hyperactivity disorder (ADHD) or weight management. With the continuation of the COVID-19 pandemic, individuals who previously recovered from COVID-19 can become re-infected. Repeated SARS-CoV-2 infections can increase the risk of adverse health outcomes, especially among persons already experiencing chronic physical and mental health problems.^2^

Concerns have also been raised that the use of stimulants could contribute to the development of substance use disorders (SUDs)^3^ and adverse cardiovascular effects^4,5^ that, in turn, have been associated with a greater risk of COVID-19 infection and worse outcomes.^6^ Moreover, adults prescribed stimulants often experience obesity, diabetes, and mental health comorbidities^4,7^ that have been found to negatively influence immune and respiratory systems, increasing vulnerability to the SARS-CoV-2 virus infection^8^ and its complications.^9-11^

Yet, stimulant medications are an evidence-based pharmacotherapy for ADHD,^12,13^ and prescribed for weight loss.^14,15^ The number of new prescriptions for stimulants increased by 250% in the U.S. from 2006-2020,^7^ and approximately 55% of all stimulant prescriptions have been issued to adults annually since 2015.^16^ Previous research from secondary database analysis using large existing electronic health record (EHR) data found that adults on stimulant therapy were associated with the lower rate of emergency room visits, hospital admissions, intensive care unit utilization, and deaths within 30 days of initial infection.^17^ Yet, little is known about the potential role stimulant medications in the risk and severity of COVID-19 reinfection, despite the fact the stimulants are used to treat common risk factors for COVID-19 harms (i.e., mental health disorder, obesity) and may themselves result in adverse cardiovascular events.

This study aimed to evaluate the risk of COVID-19 reinfection and its severe adverse outcomes among adults prescribed stimulants compared to those not on stimulant therapy. Elucidating the potential impact of COVID-19 reinfection risk and harm for those prescribed stimulant therapy can improve clinician understanding and care delivered to patients.

## 2. Methods

### 2.1 STUDY DESIGN AND DATA SOURCE

This was a retrospective cohort study utilizing electronic health records (EHRs) of 57 healthcare organizations sourced from the TriNetX research network database (Cambridge, MA). TriNetX is a global health data network that contains de-identified EHRs (demographics, diagnoses, procedures, medications, and laboratory tests) of more than 80 million patients from participating healthcare organizations from the U.S. Data in the TriNetX database has been mapped to common clinical entities and terminologies to ensure high usability as well as consistency with the Reporting of studies Conducted using Observational Routinely collected Data (RECORD) guidelines.^18^ The study used deidentified TriNetX research datasets which were be exempt from the Institutional Review Board oversight by the Pennsylvania State University’s Human Research Protection Program.

### 2.2 STUDY POPULATION

The study population consisted of adults (age≥18 years) who were diagnosed during the study enrollment period, which spanned January 1, 2020 and April 30, 2022, with an initial COVID-19 infection (“index infection”) and belonged to one of the two participant cohorts (see below). COVID-19 reinfection was defined as a COVID-19 diagnosis reported 30 or more days after the index infection. Presence of COVID-19 illness was defined through the combination of COVID-19 diagnosis and positive laboratory test results^4,19^ (see Supplemental Table S1 for details). The EHR data on COVID-19-relevant outcomes were collected continuously through July 31,2022, allowing the analysis to capture COVID-19 reinfection data after the study enrollment period. Individuals were excluded if they had cancer diagnoses or received skilled nursing facility or palliative care prior to their index COVID-19 infection.

Two cohorts were identified within the study population. The ‘stimulant cohort’ consisted of adults with at least three prescriptions for stimulants and the ‘control cohort’ included those who did not receive any stimulant prescriptions within six months prior to their index COVID-19 infection. Therefore, for persons with COVID-19 diagnosed within the first few months of the study enrollment period, stimulant medications could have been prescribed before January 1, 2020.

Stimulant medications were identified based on both generic (amphetamine, dextroamphetamine, lisdexamfetamine, methylphenidate, methamphetamine, dexmethylphenidate) and brand names, ^20,21^ using normalized name and code sets for medications based on the Prescription for Electronic Drug Information Exchange (RxNorm).^22^ The list of RxNorm code sets is provided in Supplemental Table S1.

### 2.3 OUTCOME MEASURES OF COVID-19 REINFECTION AND ITS SEVERITY

The COVID-19 reinfection was assessed using a binary approach (yes/no). The presence of severe COVID-19 reinfection outcomes was defined through dichotomous (yes/no) healthcare utilization and mortality metrics, including the occurrence of emergency department (ED) visits, hospital or intensive care unit (ICU) admissions, or death within 30 days of the reinfection date.

### 2.4 BASELINE CHARACTERISTICS

Data on participant demographics and selected health comorbidities were extracted at the time of the index COVID-19 infection. The available demographic data included sex (male orfemale), age category (18-39, 40-64, or 65+ years old), ethnicity (Hispanic or non-Hispanic) and race (White, Black or Other). Data on health conditions known to increase the risk of COVID-19 complications were also collected, including the diagnoses of chronic medical conditions (diabetes, obesity/overweight, cardiovascular disease, chronic kidney disease, chronic liver disease, chronic lung disease, immune problems, sickle cell disease),^11,19^ mental health disorders (anxiety, bipolar disorder, dementia, depression, ADHD),^23^ alcohol/drug use disorders (alcohol, opioid, cocaine, stimulant, and cannabis use disorders) and tobacco/nicotine use disorder, with the latter serving as a proxy for smoking/tobacco use status, which is unavailable within the TriNetX data. Detailed information on the diagnostic codes for the conditions extracted in this study is provided in Supplemental Table S1.

### 2.5 STATISTICAL ANALYSIS

Descriptive statistics were computed to summarize sample characteristics and each outcome measure. Differences in continuous variables were compared using the t-test for parametric or equivalent tests for non-parametric data. Proportion differences in categorical variables were evaluated using the Chi-square test. Multiple logistic regression modeling was used to assess the risk of reinfection as well as severe COVID-19-related complications of, including ED visit, hospitalization, ICU admission and death within 30 days of COVID-19 reinfection (binary dependent variables, Yes/No), controlled for baseline demographics and comorbid conditions known to contribute to the COVID-19 severity risk. The regression analyses were conducted using the Maximum Likelihood Estimation method, which provided regression coefficients, standard errors (SEs), Wald 95% confidence intervals (Cis) for the coefficients, and p-values for each of the model variables. The adjusted odds ratio (aOR) and 95% Cl of each variable was also calculated to predict the risk of the outcome measure. The significance level was determined based on two-tailed p-value < 0.05.

Moreover, we conducted sensitivity analyses with respect to patients filled at least 9 stimulant prescriptions within one year prior to their index COVID-19 infection to evaluate for possible cumulative effect of the stimulant treatment on the subsequent risk of COVID-19 reinfection and severe outcomes. Statistical analyses were performed using PROC LOGISTIC procedure (Version 9.4 SAS Institute Inc., Carey, NC).

## 3. Results

### 3.1 STUDY SAMPLE

**Figure 1.**
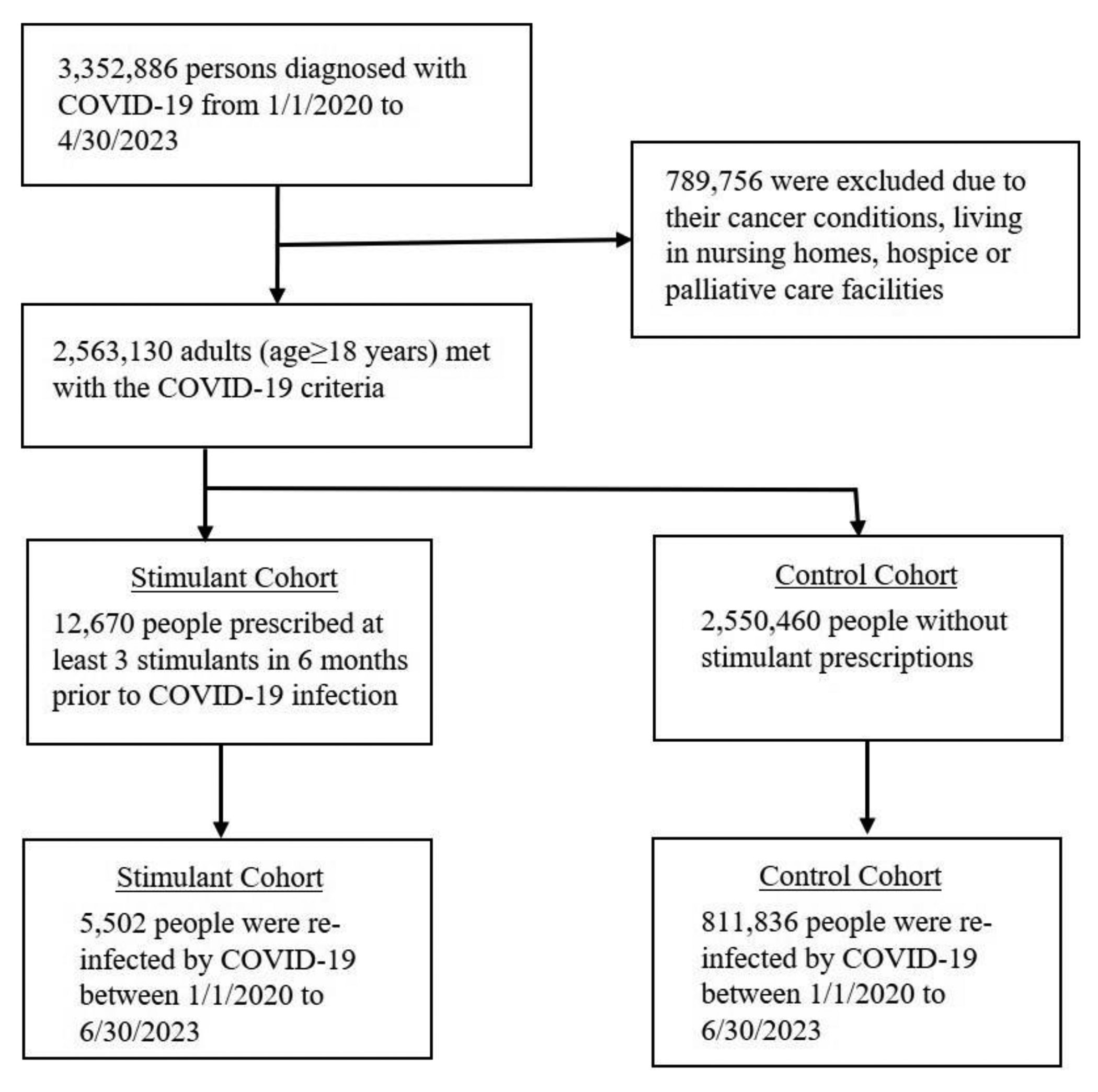
Flowchart of the study cohort selection process.

The study sample consisted of 2,563,130 adults diagnosed with COVID-19 between January 1, 2020 and April 30, 2022, including 12,670 persons in the stimulant cohort and 2,550,460 in the control cohort. The flow diagram of the cohort selection process is provided in Figure 1. Overall, the stimulant cohort had a greater percentage of females and Whites, was slightly younger, and had higher rates of chronic medical/mental health conditions, tobacco/nicotine as well as alcohol/drug use disorders, known risk factors for COVID-19 complications (Table 1).

### 3.2 RISK OF COVID-19 REINFECTION AND SEVERE COMPLICATIONS

Close to one-half (43.4%) of the stimulant cohort participants had a documented COVID-19 reinfection, compared to 31.8% of the control cohort (Table 2). Among those with COVID-19 reinfection, individuals in the stimulant cohort, compared to the control cohort, showed greater frequency (p<0.01) of the ED, hospital and ICU care, and mortality within 30 days of reinfection.

**Figure 2.**
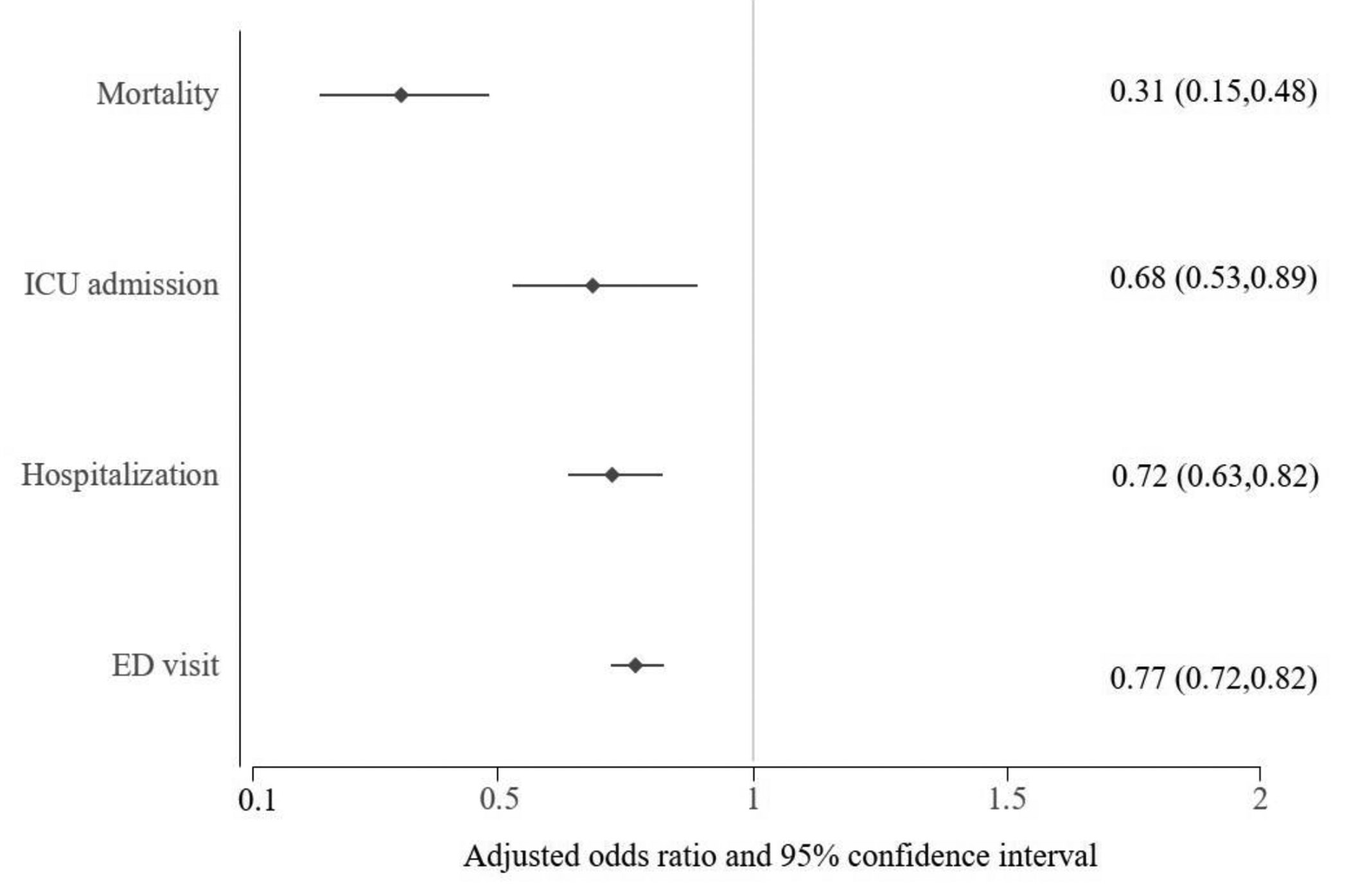
Logistic regression analysis: The Likelihood of COVID-19 reinfection among the entire study Sample (N=2,563,130) and the risk of Severe complications among those with COVID-19 reinfection (N=817,338) among adults within the stimulant cohort compared to those in the control cohort

The logistic regression analysis, controlled for baseline characteristics, revealed that individuals in the stimulant cohort, compared to those in the control cohort, were 1.7 times more likely to be reinfected by COVID-19 (aOR=1.66, 95% Cl: 1.60, 1.72) (Figure. 2). However, once reinfected, the stimulant cohort was found to have lower risk of requiring ED (aOR=0.77, 95% Cl: 0.72, 0.82), hospital (aOR=0.72, 95% Cl: 0.63, 0.82) or ICU (aOR=0.68, 95% Cl: 0.53, 0.89) care, or dying (aOR=0.31, 95% Cl: 0.15, 0.48) within 30 days of reinfection. The details of the statistical models and results for each outcome measure are provided in Supplemental Table S2.

Our sensitivity analyses focusing on adults on long-term stimulants (≥9 prescriptions in 12 months) showed that adjusted odds of COVID-19 reinfection were 1.4 times greater than those in the control cohort (aOR=1.35, 95% Cl: 1.30, 1.40). They were less likely to be admitted to ED (aOR=0.77, 95% Cl: 0.72, 0.82), hospital (aOR=0.72, 95% Cl: 0.63, 0.82) or ICU (aOR=0.68, 95% Cl: 0.53, 0.89), or die (aOR=0.31,95% Cl: 0.15, 0.65) within 30 days of reinfection. The findings of the sensitivity analyses were consistent to the results of the primary analyses.

## 4. Discussion

### 4.1 COVID-19 REINFECTION

Our findings, based on a secondary database analysis of large existing EHRs, found that, in general, adults treated with stimulant therapy were more likely to experience a COVID-19 reinfection compared to those not prescribed stimulants. These findings are consistent with previous EHR-based research, which showed higher overall COVID-19 infection risk among patients with ADHD who were treated with stimulants compared to those without this treatment,^24^ suggesting that adults prescribed stimulants might be more susceptible to COVID-19 reinfection.

The higher reinfection rate could be possibly contributed by multiple factors. For instance, a substantial portion of adults in the stimulant cohort were young and working adults. Those individuals often faced elevated COVID-19 reinfection risks due to close physical proximity and high interaction with other people at work or school. Moreover, a greater percentage of the stimulant cohort had chronic conditions, such as obesity, diabetes, cardiovascular disease, and chronic kidney disease, which have been identified as risk factors for COVID-19 infection.^8,10^

### 4.2 RISK OF SEVERE COMPLICATIONS AFTER REINFECTION

Interestingly, our analyses showed that, despite a higher COVID-19 reinfection risk, adults on stimulant therapy displayed a lower risk of severe outcomes and morality within 30 days after reinfection. These findings are aligned with prior literature showing that treatment with stimulants was associated with fewer severe COVID-19 complications.^17^ The lower risk of complications could be, at least partially, explained by presumed higher access to and engagement in care among stimulant-treated individuals, as regular office visits are a part of standard of care when prescribing long-term stimulants and other controlled substances.^25,26^ Moreover, stimulant therapy aims to improve executive function, such as self-control and motivation,^27,28^ which, in turn, could help increase awareness of and adherence to public health recommendations aimed at reducing the risk of infection and harms associated with COVID-19. At last, the immune system of people with repeated COVID-19 infection might help protect them from severe illness, though the level of protection could depend on virus variants, comorbid conditions, and other factors.^290^

The strengths of this study included analyses leveraging large EHR research database, which included over 2.6 million adults with COVID-19 infection in the U.S. The use of a large database allowed for evaluation of COVID-19 reinfection among individuals prescribed stimulants based on existing longitudinal, real-life EHR data and adjust for the potential impacts of demographic and relevant clinical factors, offering additional insights into the potential risks versus benefits of stimulant therapy.

### 4.3 STUDY LIMITATIONS

There are several limitations of this study. The COVID-19 diagnosis was established based on the data shared with TriNetX by the collaborating health systems; therefore, such data would not be available from COVID-19 diagnosis or testing done outside of the participating data network. This could have underestimated the prevalence of COVID-19 reinfection, particularly within the control cohort whose members would be anticipated to be more likely to seek acute care outside of the participating networks rather than those in the stimulant cohort whose stimulant therapy had been managed by the network’s clinicians. Second, the research data did not contain information related to patients’ socioeconomic contexts (e.g., insurance, education and income levels); future studies accounting for socioeconomic potential confounders could help further our understanding of most salient risk versus protective factors. Although literature has documented that COVID-19 vaccines reduce the risk of infection and severe illness,^30,31^ we were unable to account for the potential impact of this preventive strategy due to fewer than 10% of participants with a recorded receipt of their COVID-19 vaccine. This issue is likely due to the fact that vaccination efforts, especially early during the pandemic, had been carried out largely by public health, then pharmacies, rather than health systems, with a resulting underreporting of the COVID-19 vaccination in the EHR. With such low prevalence of COVID-19 vaccine receipt noted within our sample, we did not believe the vaccine effect could be meaningfully assessed within the study sample. Yet, our exploratory analysis showed similar findings to those reported in this manuscript even after controlling for the vaccine receipt, supporting the validity of our overall findings. Additional research using a prospective design to understand the demographic and clinical underpinnings of COVID-19 reinfection and its severe outcomes is warranted.

## 5. Conclusion

The COVID-19 pandemic has caused devastating impacts within our communities, especially among populations with chronic medical or mental health conditions. Our study found that adults on stimulant therapy were more likely to be re-infected by COVID-19, but had lower risk of severe illness and mortality within 30 days of reinfection. The big data analytics developed in this study offers an innovative approach to routinely, efficiently assess the impact of COVID-19 and other emerging treatments and new public health threats, and help clinicians make informed decisions when treating patients with stimulant medications.

## Supplementary Material

Supplemental materias

## Figures and Tables

**Table 1.** Baseline characteristics of the study sample of adults with COVID-19 infection by cohort status.

Characteristics, n (%)	Total, N=2,563,130	Stimulant Cohort, N=12,670	Control Cohort, N=2,550,460	p-value^†^
Female	1,415,416(55.2)	7,765 (61.4)	1,407,651 (55.2)	<0.01
Age category				
18-39	1,112,815 (43.4)	8,165 (64.4)	1,104,650 (43.3)	<0.01
40-64	1,072,012 (41.8)	4,215 (33.3)	1,067,797 (41.9)	
65+	378,303 (14.8)	290 (2.3)	378,013 (14.8)	
Hispanic	174,759 (6.8)	646 (5.1)	174,113 (6.8)	<0.01
Race				<0.01
White	1,152,850 (45)	10,617 (83.8)	1,142,233 (44.8)	
Black	344,969 (13.5)	819 (6.5)	344,150 (13.5)	
Other	1,065,311 (41.6)	1,234 (9.7)	1,064,077 (41.7)	
Medical/mental health conditions				
Diabetes	193,042 (7.5)	786 (6.2)	192,256 (7.5)	<0.01
Obesity/overweight	263,625 (10.3)	2,503 (19.8)	261,122 (10.2)	<0.01
Cardiovascular disease	464,544 (18.1)	2,802 (22.1)	461,742 (18.1)	<0.01
Chronic kidney disease	73,642 (2.9)	396 (3.1)	73,246 (2.9)	<0.01
Chronic liver disease	13,015 (0.5)	90 (0.7)	12,925 (0.5)	<0.01
Chronic lung disease	232,915 (9.1)	2,860 (22.6)	230,055 (9.0)	<0.01
Immune problem	24,394 (1.0)	229 (1.8)	24,165 (0.9)	<0.01
Sickle cell disease	5,483 (0.2)	20 (0.2)	5,463 (0.2)	<0.01
Mental health problem^1^	345,438 (13.5)	7,370 (58.2)	338,068 (13.3)	<0.01
Alcohol use disorder	35,442 (1.4)	428 (3.4)	35,014 (1.4)	<0.01
Nicotine/tobacco use disorder	139,357 (5.4)	1,446 (11.4)	137,911 (5.4)	<0.01
Drug use disorders^2^	47,931 (1.9)	832 (2.3)	22,276 (1.1)	<0.01

† Chi-square statistics were computed to evaluate differences in proportions between the stimulant and control cohorts.

1 Mental health problem included anxiety, bipolar disorder, dementia, depression, and ADHD.

2 Drug use disorders includes opioid, cannabis, sedative, cocaine, other stimulant, hallucinogen, inhalant, or psychoactive drug use disorders, and excludes alcohol and nicotine/tobacco use disorders

**Table 2.** COVID-19 Reinfection and its Complications: Frequency across the Study Cohorts.

Characteristics, n (%)	Total, N=2,563,130	Stimulant Cohort, N=12,670	Control Cohort, N=2,550,460	p-value^†^
Reinfection	817,338 (31.9)	5,502 (43.4)	811,836 (31.8)	<0.01
Among those with re-infection:				
ED visit, Yes	157,000 (6.1)	1,232 (9.7)	155,768 (6.1)	<0.01
Hospitalization, Yes	46,427 (1.8)	258 (2.0)	46,169 (1.8)	<0.01
ICU admission, Yes	13,575 (0.5)	58 (0.5)	13,517 (0.5)	<0.01
Death, Yes	6,138 (0.2)	7 (0.1)	6,131 (0.2)	<0.01

ED: Emergency department; ICU: Intensive care unit

† Chi-square statistics were computed to evaluate differences in proportions between the stimulant and control cohorts.
