# Supplementary material for: Assessing risk of COVID-19 reinfection and complications in adults treated with stimulant medications: A retrospective study using electronic health records
Source: Med Res Arch. Author manuscript; Available in PMC 2026 Jun 27. (PMC13309181; doi:10.18103/mra.v12i9.5807)
Supplement: Supplemental materias [file NIHMS2186584-supplement-Supplemental_materias.docx]

**Supplemental Materials**

**Table S.1.** Description of coding systems and codes

| Coding System | Code | Description |  |
| --- | --- | --- | --- |
| *COVID-19 diagnoses and lab tests* | | |  |
| ICD-10 | U07.1, U07.2 | COVID-19 (WHO) |  |
| ICD-10 | B97.29 | Other coronavirus as the cause of diseases classified elsewhere |  |
| ICD-10 | B34.2 | Coronavirus infection, unspecified |  |
| ICD-10 | J12.81 | Pneumonia due to SARS-associated coronavirus |  |
| LOINC | 94505-5,94506-3,94558-4, 94562-6, 94762-2,94769-7,95209-3 | SARS-CoV-2 (COVID19) [presence] in serum or plasma by immunoassay |  |
| *Prescription stimulants* | | |  |
| RxNorm | 725,3288,6816,6901,352372,700810 | Stimulant medication |  |
| *Baseline characteristics* | | |  |
| ICD-10 | E10-E11 | Diabetes (type I and II) |  |
| ICD-10 | E66 | Obesity/overweight, |  |
| ICD-10 | I10, I20, I21, I22, I23, I24, I25, I26, I27, I28, I48, I49, I50, I63 | Cardiovascular disease |  |
| ICD-10 | N18 | Chronic kidney diseases |  |
| ICD-10 | K70, K73, K74, K721, K754 | Chronic liver diseases |  |
| ICD-10 | J40, J41, J42, J43, J44, J45, J46, J47, J44, E84, A15 | Chronic lung diseases |  |
| ICD-10 | B20, D849, T86, Z94 | Immune problem |  |
| ICD-10 | D56, D57 | Sickle cell disease | |
| ICD-10 | F03, F31, F32, F33, F41, F90 | Mental health disorders |  |
| ICD-10 | F17.1, F17.2 | Tobacco/nicotine dependence | |
| ICD-10 | F10.1, F10.2 | Alcohol use disorder |  |
| ICD-10 | F11.1, F11.2, F12.1, F12.2, F13.1, F13.2, F14.1, F14.2, F15.1, F15.2, F16.1, F16.2,  F18.1, F18.2, F19.1, F19.2 | Drug use disorders involving cannabis, sedatives, cocaine, stimulants, hallucinogens, inhalants or other psychoactive drugs (, excluding alcohol and nicotine/tobacco) |  |

**Table S2.** The likelihood of COVID-19 reinfection and related complications within 30 days of COVID-19 reinfection.

|  |  | Outcomes within 30 days of COVID-19 reinfection^‡^ | | | |
| --- | --- | --- | --- | --- | --- |
| Factors | Reinfection^†^,  aOR (95%CI) | Emergency Department,  aOR (95%CI) | Hospitalization,  aOR (95%CI) | Intensive Care,  aOR (95%CI) | Death,  aOR (95%CI) |
| Stimulant therapy | 1.36^**^ (1.32,1.41) | 0.77^**^ (0.72,0.82) | 0.72^**^ (0.63,0.82) | 0.68^**^ (0.53,0.89) | 0.31^**^ (0.15,0.64) |
| Female | 1.23^**^ (1.23,1.24) | 1.06^**^ (1.05,1.07) | 0.85^**^ (0.83,0.86) | 0.68^**^ (0.66,0.71) | 0.67^**^ (0.64,0.71) |
| Age (ref: 18-39 yrs) |  |  |  |  |  |
| 40-64 yrs | 0.85^**^ (0.84,0.85) | 0.82^**^ (0.81,0.83) | 1.27^**^ (1.24,1.31) | 1.78^**^ (1.69,1.87) | 4.14^**^ (3.70,4.64) |
| 65+ yrs | 0.71^**^ (0.70,0.71) | 1.13^**^ (1.11,1.15) | 2.92^**^ (2.83,3.01) | 3.26^**^ (3.09,3.45) | 19.43^**^ (17.34,21.74) |
| Hispanic | 0.81^**^ (0.80,0.82) | 2.43^**^ (2.38,2.48) | 2.45^**^ (2.37,2.53) | 2.03^**^ (1.91,2.16) | 1.46^**^ (1.31,1.61) |
| Race (ref: White) |  |  |  |  |  |
| Black | 0.96^**^ (0.96,0.97) | 2.20^**^ (2.17,2.24) | 1.33^**^ (1.30,1.36) | 1.36^**^ (1.31,1.43) | 1.23^**^ (1.15,1.31) |
| Other | 1.12^**^ (1.11,1.12) | 0.28^**^ (0.27,0.28) | 0.29^**^ (0.28,0.29) | 0.38^**^ (0.36,0.40) | 0.41^**^ (0.38,0.45) |
| Chronic health conditions |  |  |  |  |  |
| Diabetes | 0.96^**^ (0.95,0.97) | 1.37^**^ (1.34,1.40) | 1.53^**^ (1.49,1.57) | 1.69^**^ (1.61,1.76) | 1.47^**^ (1.38,1.56) |
| Obesity/overweight | 1.10^**^ (1.09,1.11) | 1.02^**^ (1.01,1.04) | 0.99 (0.97,1.02) | 0.91^**^ (0.87,0.95) | 0.80^**^ (0.75,0.86) |
| Cardiovascular disease | 1.09^**^ (1.08,1.10) | 1.50^**^ (1.48,1.53) | 1.82^**^ (1.77,1.86) | 2.06^**^ (1.97,2.16) | 1.58^**^ (1.47,1.69) |
| Chronic kidney disease | 1.24^**^ (1.22,1.26) | 1.30^**^ (1.27,1.34) | 1.92^**^ (1.86,1.98) | 1.95^**^ (1.85,2.05) | 2.59^**^ (2.43,2.77) |
| Chronic liver disease | 1.38^**^ (1.33,1.43) | 1.40^**^ (1.32,1.49) | 1.99^**^ (1.87,2.14) | 2.20^**^ (2.01,2.40) | 2.39^**^ (2.11,2.71) |
| Chronic lung disease | 1.26^**^ (1.25,1.27) | 1.43^**^ (1.41,1.46) | 1.21^**^ (1.18,1.24) | 1.30^**^ (1.24,1.35) | 1.28^**^ (1.20,1.36) |
| Immune problem | 1.36^**^ (1.33,1.40) | 0.89^**^ (0.85,0.93) | 1.39^**^ (1.32,1.47) | 1.12^**^ (1.02,1.22) | 1.32^**^ (1.17,1.49) |
| Sickle cell disease | 1.33^**^ (1.27,1.39) | 1.19^**^ (1.10,1.28) | 2.49^**^ (2.27,2.73) | 1.59^**^ (1.34,1.90) | 1.67^**^ (1.23,2.27) |
| Mental health disorder^1^ | 1.21^**^ (1.20,1.22) | 1.38^**^ (1.36,1.41) | 1.25^**^ (1.22,1.28) | 1.12^**^ (1.07,1.16) | 1.10^**^ (1.04,1.17) |
| Alcohol use disorder | 1.14^**^ (1.11,1.17) | 1.683^**^ (1.62,1.75) | 1.80^**^ (1.72,1.89) | 1.80^**^ (1.68,1.94) | 1.49^**^ (1.32,1.68) |
| Nicotine/tobacco use disorder | 1.10^**^ (1.09,1.11) | 1.93^**^ (1.89,1.97) | 1.57^**^ (1.52,1.62) | 1.65^**^ (1.57,1.73) | 1.23^**^ (1.14,1.34) |
| Drug use disorder^2^ | 1.04^**^ (1.01,1.07) | 1.80^**^ (1.70,1.89) | 1.59^**^ (1.47,1.72) | 1.54^**^ (1.36,1.75) | 1.18^**^ (0.95,1.46) |

^*^*p*<0.05; ^**^*p*<0.01;

aOR: Adjusted odds ratio; 95% CI: 95% conference intervals

^†^ among the study sample, which included 12,670 adults in the Stimulant Cohort and 2,550,460 adults in the Control Cohorts

^‡^ among individuals with COVID-19 reinfection: 5,502 in the Stimulant and 811,836 in the Control Cohorts

^1^Mental health problem included anxiety, bipolar disorder, dementia, depression, and ADHD.

^2^Other drug use disorders included cannabis, sedative, cocaine, stimulant, hallucinogen, inhalant, or psychoactive drug use disorders, and excluded opioid, alcohol, and nicotine/tobacco use disorders
